# Hoveniae Semen Seu Fructus Ethanol Extract Exhibits Anti-Inflammatory Activity *via* MAPK, AP-1, and STAT Signaling Pathways in LPS-Stimulated RAW 264.7 and Mouse Peritoneal Macrophages

**DOI:** 10.1155/2019/9184769

**Published:** 2019-09-04

**Authors:** Yun Hee Jeong, You-Chang Oh, Won-Kyung Cho, Nam-Hui Yim, Jin Yeul Ma

**Affiliations:** Korean Medicine (KM)-Application Center, Korea Institute of Oriental Medicine, 70, Cheomdanro, Dong-gu, Daegu 41062, Republic of Korea

## Abstract

Hoveniae semen seu fructus (HSF, fruit and seed of *Hovenia dulcis* Thunb) is an important traditional herbal medicine and food supplement in East Asia for the treatment of liver diseases, alcohol poisoning, obesity, allergy, and cancer. HSF has also been reported to have anti-inflammatory activity, but the cellular mechanism of action is not fully understood. We assessed the anti-inflammatory properties of an HSF ethanol (HSFE) extract and explored its precise mechanism. The ability of HSFE to suppress inflammatory responses was investigated in a murine macrophage cell line, RAW 264.7, and mouse primary macrophages. Secretions of NO, proinflammatory cytokines, inflammatory factors, and related proteins were measured using the Griess assay, ELISA, Western blot analysis, and real-time PCR, respectively. In addition, the main components of HSFE were analyzed by HPLC, and their anti-inflammatory activity was confirmed. Our results showed that pretreatment of HSFE markedly reduced the expression of NO and iNOS without causing cytotoxicity and significantly attenuated secretion of proinflammatory cytokines, including TNF-*α*, IL-6, and IL-1*β*. In addition, HSFE strongly suppressed phosphorylation of MAPK and decreased the activation of AP-1, JAK2/STAT, and NF-*κ*B in LPS-stimulated RAW 264.7 cells in a concentration-dependent manner. Furthermore, HSFE strongly suppressed the inflammatory cytokine levels in mouse peritoneal macrophages. Also, as a result of HPLC analysis, three main components, ampelopsin, taxifolin, and myricetin, were identified in the HSFE extract, and each compound effectively inhibited the secretion of inflammatory mediators induced by LPS. These findings show that HSFE exerts anti-inflammatory effects by suppressing the activation of MAPK, AP-1, JAK2/STAT, and NF-*κ*B signaling pathways in LPS-stimulated macrophages. In addition, the anti-inflammatory efficacy of HSFE appears to be closely related to the action of the three main components. Therefore, HSFE appears to be a promising candidate for the treatment of inflammatory diseases.

## 1. Introduction

The inflammatory response in the body is a defense mechanism against potentially harmful stimuli, such as injury, viral or microbial infection, and irritants [1, 2]. These inflammatory responses are involved in the activation of immune cells, such as macrophages. Macrophages have an essential role in modulating innate immune responses and inflammation [3]. During the inflammation process, lipopolysaccharide- (LPS-) stimulated macrophages secrete other proinflammatory factors, such as nitric oxide (NO), prostaglandin E_2_ (PGE_2_), and cytokines, including tumor necrosis factor- (TNF-) *α*, interleukin- (IL-) 6, and IL-1*β* [4]. LPS, known as endotoxins from the outer membranes of gram-negative bacteria, act on the toll-like receptor 4- (TLR4-) signaling pathway and elicit strong immune responses. The TLR4-signaling pathway is directly linked to the phosphorylation of the mitogen-activated protein kinase (MAPK) intercellular signaling pathway [5]. The three representative families of MAPK have been identified, including extracellular signal-regulated kinase (ERK), c-Jun NH_2_-terminal kinase (JNK), and p38, which also regulate immune-related cytotoxic mediators [6]. Upon stimulation by LPS, activated MAPKs in turn mediate several signal transducers, including nuclear factor- (NF-) *κ*B and activator protein- (AP-) 1 [7]. The transcription factors NF-*κ*B and AP-1 are closely involved in the regulation of proinflammatory mediators, such as TNF, IL-6, IL-1*β*, and inducible nitric oxide synthase (iNOS) [8]. Additionally, the Janus kinase (JAK)/signal transducer and activator of transcription (STAT) cascade is another critical signaling pathway that has an important role in immune responses and inflammation via the release of proinflammatory cytokines and growth factors [9]. Accordingly, inhibiting the MAPK, AP-1, and JAK/STAT signaling pathways can be important and effective strategies for treating inflammatory disease.

*Hovenia dulcis* Thunb is a well-known oriental raisin tree in East Asia that has traditionally been used to treat liver diseases, alcohol poisoning, obesity, allergy, and cancer [10–12]. A previous study showed that *Hovenia dulcis* Thunb extract had anti-inflammatory activity by inhibiting the NF-*κ*B pathway [13]. However, other signaling pathway mechanisms of action have not yet been investigated. Therefore, this study investigated whether hoveniae semen seu fructus ethanol (HSFE) extract exerts anti-inflammatory effects on LPS-stimulated macrophage RAW 264.7 cells via the regulation of the MAPK, AP-1, and JAK/STAT signaling pathways. In addition, it was confirmed again whether HSFE pretreatment inhibited the activation of NF-*κ*B by LPS. We also investigated the anti-inflammatory effect of HSFE on LPS-stimulated mouse primary macrophages.

## 2. Materials and Methods

### 2.1. Materials and Reagents

Dulbecco's Modified Eagle Medium (DMEM), fetal bovine serum (FBS), and antibiotics were obtained from HyClone (Logan, UT, USA). LPS, dexamethasone (Dex), bovine serum albumin (BSA), dimethyl sulfoxide (DMSO), and red blood cell (RBC) lysis buffer were purchased from Sigma-Aldrich (St. Louis, MO, USA). Enzyme-linked immunosorbent assay (ELISA) antibody sets were obtained from eBioscience (San Diego, CA, USA). Cell culture dishes and plates were purchased from SPL Life Sciences (Pocheon, Korea). A cell-counting kit (CCK) was obtained from Dojindo Molecular Technologies Inc. (Kumamoto, Japan). Various primary antibodies and horseradish peroxidase- (HRP-) conjugated secondary antibodies were purchased from Cell Signaling Technology Inc. (Boston, MA, USA) and Santa Cruz Biotechnology Inc. (Santa Cruz, CA, USA). An RNA extraction kit was obtained from iNtRON Biotechnology (Daejeon, Korea). DNA-synthesizing kits and an AccuPower® 2X GreenStar qPCR (quantitative polymerase chain reaction) Master Mix were obtained from Bioneer (Daejeon, Korea). Oligonucleotide primers for real-time reverse transcription-polymerase chain reaction (real-time PCR) were synthesized by Bioneer.

### 2.2. Preparation of HSFE

HSF was purchased as a dried herb from Yeongcheonhyundai Herbal Market (Yeongcheon, Korea) and identified by Prof. KiHwan Bae, Chungnam National University, Korea. All voucher specimens were deposited in an herbal bank at the KM-Application Center, Korea Institute of Oriental Medicine (voucher number: E126). The dried herb (30.0 g) was extracted with 390 mL of 70% ethanol in a 40°C shaking incubator (100 rpm) for 24 h. The extract was filtered through a 150 mm filter paper (Whatman, Piscataway, NJ, USA) and concentrated using a rotary vacuum evaporator (Buchi, Tokyo, Japan). Samples were then freeze dried and kept in desiccators at −20°C before use. The sample yield was 4.6780%.

### 2.3. Cell Culture and Drug Treatment

Murine macrophage RAW 264.7 cells were obtained from the Korea Cell Line Bank (KCLB, Seoul, Korea) and grown in complete DMEM. The cells were then incubated with humidified 5% CO_2_ at 37°C [14]. To stimulate the cells, 200 ng/mL LPS [14] was added at the indicated periods in the presence or absence of HSFE (10, 30, or 50 *μ*g/mL).

### 2.4. Cell Viability

Cytotoxicity induced by HSFE was analyzed using a CCK. First, RAW 264.7 macrophages were seeded into 96-well plates at a density of 5 × 10^4^ cells/well. After 18 h incubation, HSFE was added to the cells, which were incubated for 48 h at 37°C with 5% CO_2_ [15]. Treatment of CCK solution, incubation time, and analysis method were performed according to a previous study with some modifications [15].

### 2.5. Determination of NO Production

NO production was analyzed by measuring the nitrite levels in the supernatants of cultured macrophages. RAW 264.7 macrophages (5 × 10^4^ cells/well) were plated, incubated with HSFE, and stimulated with LPS for 24 h. Griess reagent treatment and the analysis method were performed according to a previous report [16]. The concentration of nitrite was calculated using sodium nitrite as the standard.

### 2.6. Cytokine Determination

To determine the effects of HSFE on production of proinflammatory cytokines, cytokine production was measured using ELISA. For ELISA, 2.5 × 10^5^ RAW 264.7 macrophages/well were seeded into 24-well plates and incubated overnight. The cells were pretreated with various concentrations of HSFE for 1 h and further challenged with LPS for an additional 24 h at 37°C with 5% CO_2_ [17]. The levels of cytokines in the supernatants were measured using ELISA antibody sets according to a previous method [17].

### 2.7. Preparation of Whole Cell, Cytosolic, and Nuclear Extracts

To obtain whole cell lysates, pellets were resuspended in a radioimmunoprecipitation assay (RIPA) lysis buffer (Millipore, Bedford, MA, USA) containing protease and phosphatase inhibitors. Cytosolic and nuclear fractions were isolated by using NE-PER™ nuclear and cytoplasmic extraction reagents (Thermo Scientific, Rockford, IL, USA) according to the procedure described by the manufacturer. The fractions were stored at −80°C before use.

### 2.8. Western Blot Analyses

Western blot analyses were performed to evaluate the effects of HSFE on the expression of each protein in the whole cell, cytosol, or nucleus. The cells were pretreated with HSFE and stimulated with LPS for the indicated times. After incubation, the cells were collected *via* scrapping and washed twice with ice-cold phosphate-buffered saline (PBS). Total protein was determined using a Bradford reagent (Bio-Rad, Hercules, CA, USA). Detailed methods and conditions of the protein analysis were followed with reference to a past study [15]. The information about the various primary and secondary antibodies used is listed in Table 1. Specific proteins were detected using the SuperSignal West Femto Chemiluminescent Substrate (Thermo Scientific). Protein levels were quantified using a ChemiDoc™ Touch Imaging System (Bio-Rad).

### 2.9. RNA Extraction and Real-Time PCR

Total cellular RNA was isolated using an easy-BLUE™ RNA extraction kit (iNtRON Biotechnology) according to the manufacturer's instructions. Total RNA (1 *μ*g) was reverse transcribed into cDNA using an AccuPower® CycleScript RT PreMix (Bioneer). The oligonucleotide primers for real-time PCR used with mouse macrophage cDNA are listed in Table 2. The reactions were performed in triplicate, with a 20 *μ*L total volume: 0.3 *μ*M final concentrations of each primer, 10 *μ*L of AccuPower® 2X GreenStar qPCR Master Mix (Bioneer), and 2 *μ*L template DNA. The following PCR conditions were applied: TNF-*α*, IL-6, IL-1*β*, iNOS, cyclooxygenase- (COX-) 2, heme oxygenase-1, and *β*-actin and 40 cycles at 94°C for 15 s and 60°C for 1 min [14]. The amplification and analyses were performed using a QuantStudio 6 Flex Real-time PCR System (Thermo Scientific). Samples were compared using the relative C_T_ method. The fold increase or decrease in gene expression was determined relative to a blank control after normalization to the *β*-actin gene using 2^–ΔΔC_T_^ [14].

### 2.10. Peritoneal Macrophage Isolation and Cell Culture

Male BALB/c mice (25 ± 3 g) were obtained from Samtako BioKorea (Osan, Korea). The mice were inoculated with 300 *μ*L of sterile 3% sodium thioglycolate (Sigma-Aldrich, St. Louis, MO, USA). All mice were housed five per cage at room temperature with a 12 h : 12 h light/dark cycle; food (SCF Co. Ltd., Korea) and water were provided *ad libitum*. After 3 days, the animals were euthanized and macrophages were harvested by washing their peritoneal cavity with 10 mL ice-cold PBS. The cell suspension was centrifuged at 500 g for 5 min at 4°C, and the supernatant was discarded. The cell pellet was diluted in RBC lysis buffer (5 mL/mouse) and incubated at room temperature for 10 min, and the supernatant was removed through centrifugation. The cell pellet was suspended in completed Roswell Park Memorial Institute (RPMI) 1640 medium and incubated for 18 h to be attached to the cell culture plate. To stimulate the cells, the medium was replaced with fresh RPMI 1640 medium, and LPS (200 ng/mL) [18] was added in the presence or absence of HSFE (10, 30, or 50 *μ*g/mL) for the indicated periods. All animal studies were performed according to the Guide for the Animal Care and Use Committee of the Korea Institute of Oriental Medicine (reference numbers #14-079).

### 2.11. Chromatographic Conditions

Standardization of HSFE was performed by high-performance liquid chromatography (HPLC) fingerprinting with chemical standards purchased from Sigma-Aldrich. A standard stock solution containing ampelopsin, taxifolin, and myricetin was dissolved in 1 mg/mL methanol, and HSFE was weighed accurately and dissolved in 20 mg/mL methanol for analysis. All samples were filtered through a 0.2 *μ*m syringe membrane filter (Whatman Ltd., Maidstone, UK) before injection into the HPLC system for analysis.

The analytical HPLC data were obtained using a Dionex UltiMate 3000 system equipped with a binary pump, autosampler, column oven, and diode array UV/VIS detector. The output signal of the detector was recorded using Chromeleon software for the HPLC system. The analytical HPLC column used in this study was Dionex Acclain®120 C_18_ (4.6 × 150 mm, 5 *μ*m, Dionex Co., CA, USA). HPLC analysis of HSFE was performed in accordance with the methods previously reported by Park et al. [19] with some modifications. In brief, the injection volume of the sample was 10 *μ*L, and the column temperature was maintained at 40°C. The mobile phase consisted of water containing (A) 0.1% TFA and (B) acetonitrile with gradient elution at a flow rate of 0.8 mL/min. Gradient elution was as follows: 20% (*v*/*v*) B at 0–3 min, 20%–30% B at 3–15 min, 30%–90% B at 15–18 min, and 90% B at 18–20 min. The identification of the peaks was based on the UV spectrum and retention time of each marker component from the HSFE extract.

### 2.12. Preparation of the Main Components of HSFE for Cell Application

To confirm the anti-inflammatory activity of the main components of HSFE identified by HPLC analysis in macrophages, each compound was dissolved in 100% DMSO. The stock concentrations of the three compounds were 20 mM (100% DMSO), and the intracellular application concentrations were 1, 10, and 50 *μ*M, respectively (DMSO 0.25% or less). Using these compounds, the effect on the cell viability in RAW 264.7 macrophages was measured and the inhibitory effect on the secretion of NO and inflammatory cytokine by LPS was confirmed.

### 2.13. Statistical Analysis

The results are expressed as the mean ± standard error of the mean (SEM) for all experiments, and all quantitative data are representative of at least three independent experiments. Student's *t-*test was used to determine the statistically significant difference between the control or each treated group and the negative control (LPS). ^#^*P* < 0.001 (vs. control) and ^∗∗^*P* < 0.001 (vs. LPS) values were considered as indicating statistical significance.

## 3. Results

### 3.1. HSFE Treatment Did Not Cause Cytotoxicity in RAW 264.7 Macrophages

**Figure 1:**
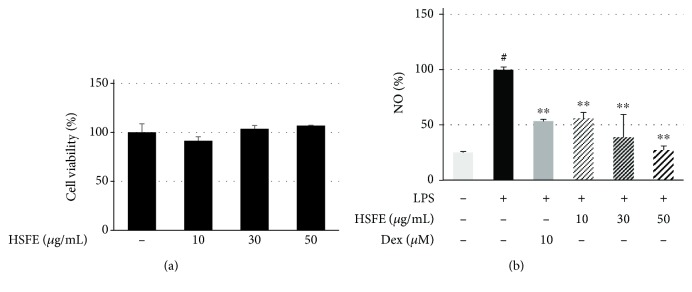
Effects of hoveniae semen seu fructus ethanol (HSFE) extract on (a) cell viability and (b) secretion of NO in macrophages. Cells were seeded with 5.0 × 10^4^ cells/well on a 96-well culture plate and preincubated for 18 h. Then, cells were pretreated with HSFE for 1 h prior to 24 h incubation with LPS. At least three independent tests were repeated to ensure reproducibility of the experimental results. (a) Cell viability was examined using a cell-counting kit. (b) NO secretion into the culture media was determined using the Griess assay. As a control, cells were incubated with the vehicle alone. Data represent the mean ± SEM of duplicate determinations from three independent experiments. ^#^*P* < 0.001 (vs. control) and ^∗∗^*P* < 0.001 (vs. LPS) values were considered statistically significant.

The cell viability test of RAW 264.7 macrophages was performed using a CCK after 48 h of HSFE treatment. The treatment did not affect the cell viability of macrophages at concentrations > 50 *μ*g/mL (Figure 1(a)). Therefore, HSFE was used at a concentration of ≤50 *μ*g/mL in the subsequent experiments.

### 3.2. Inhibitory Effect of HSFE on LPS-Induced NO Secretion and Expression of iNOS and COX-2 in RAW 264.7 Macrophages

**Figure 2:**
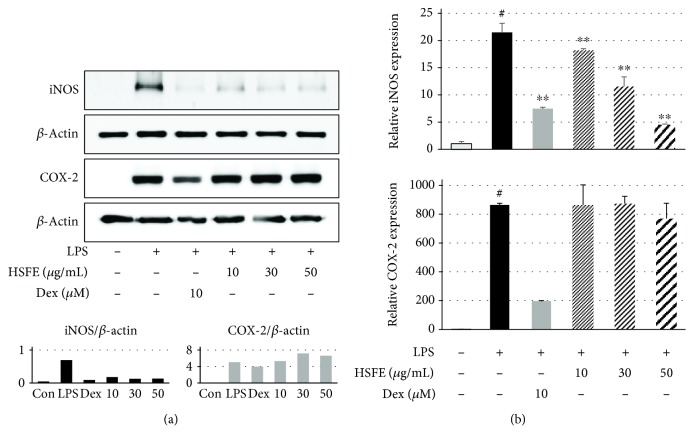
Effects of hoveniae semen seu fructus ethanol (HSFE) extract on (a) iNOS and COX-2 proteins and (b) their mRNA gene expressions. Cells were seeded with 1.5 × 10^6^ cells/well on a 6-well culture plate and preincubated for 18 h. Then, macrophages were stimulated with LPS for 24 h after 1 h pretreatment with HSFE. At least three independent tests were repeated to ensure reproducibility. Data in the histograms show protein or mRNA expression levels relative to those of *β*-actin. The experiment was repeated three times independently, and similar results were obtained. Data represent the mean ± SEM from three independent experiments. ^#^*P* < 0.001 (vs. control) and ^∗∗^*P* < 0.001 (vs. LPS) values were considered statistically significant. Con: control.

We used the Griess assay to investigate whether HSFE treatment regulates NO secretion in LPS-stimulated macrophages. Dex, which is widely used as an anti-inflammatory agent, was used as a positive control in all the HSFE pharmacological activity tests, including this experiment. The secretion of NO caused by LPS stimulation was markedly inhibited by HSFE and Dex pretreatment and showed a concentration-dependent inhibitory effect, and at 30 and 50 *μ*g/mL HSFE, the inhibitory activity was superior to that of the positive control drug (Figure 1(b)). We also measured the protein and gene expressions of iNOS and COX-2, the synthetic enzymes of NO and PGE_2_, respectively, by Western blot and real-time PCR. iNOS and COX-2 protein expressions were significantly increased by LPS stimulation (Figure 2(a)), and HSFE pretreatment showed strong, dose-dependent inhibition of iNOS, whereas COX-2 was not inhibited at all. In addition, HSFE strongly inhibited iNOS mRNA in a dose-dependent manner with statistical significance, but did not inhibit COX-2 mRNA in any way, showing a pattern similar to the inhibition of protein expression (Figure 2(b)).

### 3.3. Effects of HSFE on Secretion of TNF-*α*, IL-6, and IL-1*β* Cytokines and Their mRNA Gene Expressions

**Figure 3:**
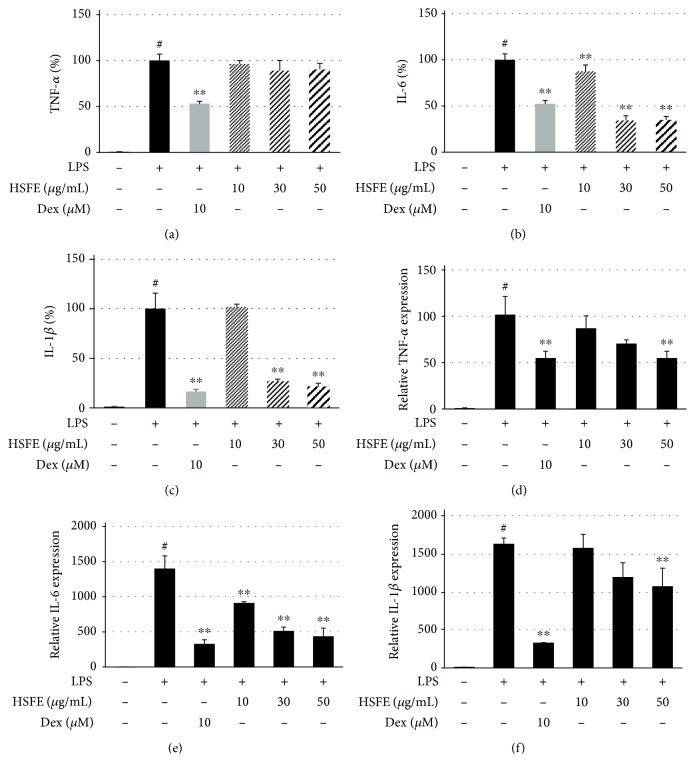
Effects of hoveniae semen seu fructus ethanol (HSFE) extract on (a–c) production of inflammatory cytokines and (d–f) their mRNA expressions after LPS stimulation of macrophages. Cells were seeded with (a–c) 2.5 × 10^5^ cells/well on a 24-well culture plate or (d–f) 1.5 × 10^6^ cells/well on a 6-well culture plate and preincubated for 18 h. Then, cells were pretreated with HSFE for 1 h and stimulated with LPS for another (a–c) 24 h or (d–f) 6 h. At least three independent tests were repeated to ensure reproducibility. Data represent the mean ± SEM of duplicate determinations from three independent experiments. ^#^*P* < 0.001 (vs. control) and ^∗∗^*P* < 0.001 (vs. LPS) values were considered statistically significant.

Since the expressions of proinflammatory cytokines by specific stimuli sources, such as LPS, are closely related to the elevation of various acute and chronic inflammatory diseases, we investigated the effect of HSFE on production of different inflammatory cytokines by performing an ELISA and a real-time PCR assay. In Figures 3(a)–3(c), HSFE pretreatment showed little inhibitory effect on TNF-*α* cytokine production and effectively inhibited the cytokines IL-6 and IL-1*β*. In parallel, each cytokine mRNA gene exhibited similarly superior and dose-dependent inhibition by HSFE treatment; in particular, TNF-*α* mRNA exhibited better inhibition than did TNF-*α* cytokine (Figures 3(d)–3(f)).

### 3.4. Inhibitory Effect of HSFE Pretreatment on Phosphorylation of the MAPK Protein

**Figure 4:**
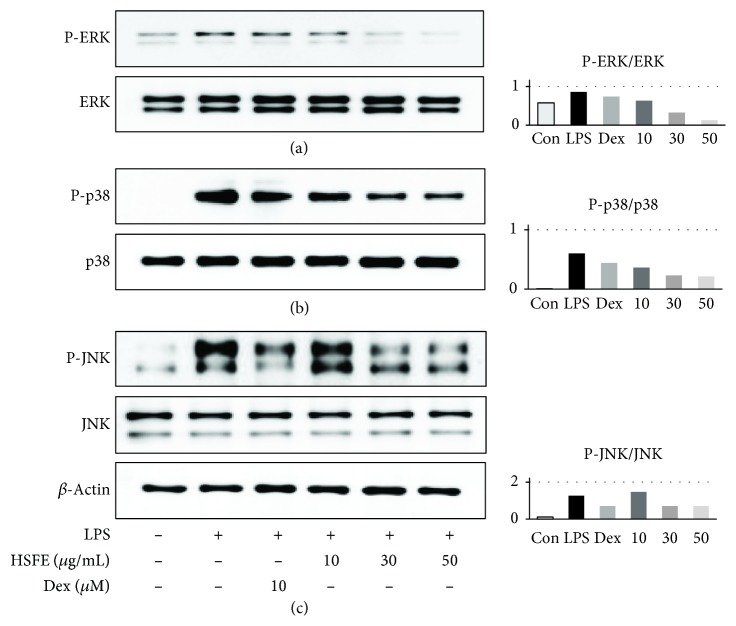
Effects of hoveniae semen seu fructus ethanol (HSFE) extract on phosphorylation of (a) ERK, (b) p38, and (c) JNK mitogen-activated protein kinases in macrophages. Cells were seeded with 1.5 × 10^6^ cells/well on a 6-well culture plate and preincubated for 18 h. Then, cells were treated with HSFE for 1 h and stimulated with LPS for another 30 min. At least three independent tests were repeated to ensure reproducibility. Total ERK, p38, and JNK served as the controls for their phosphorylated forms. Data in the histograms show protein expression levels relative to those of total-type protein. The results shown are representative of three separate experiments. Con: control.

MAPK is an important signaling pathway for regulation of the immune response and inflammatory factor expression and has an important role in regulating NF-*κ*B activation, and phosphorylation of MAPK is closely related to modulation of AP-1 activation. We therefore investigated the effect by pretreatment of HSFE on activation of the MAPK proteins ERK, p38, and JNK on LPS stimulation. Western blot analysis indicated that HSFE showed dramatic and dose-dependent attenuation of phosphorylation of three proteins at concentrations > 30 *μ*g/mL and did not affect the amount of each total-form protein (Figure 4).

### 3.5. HSFE Pretreatment Has an Inhibitory Effect on LPS-Induced AP-1 Signaling Pathway Activation

**Figure 5:**
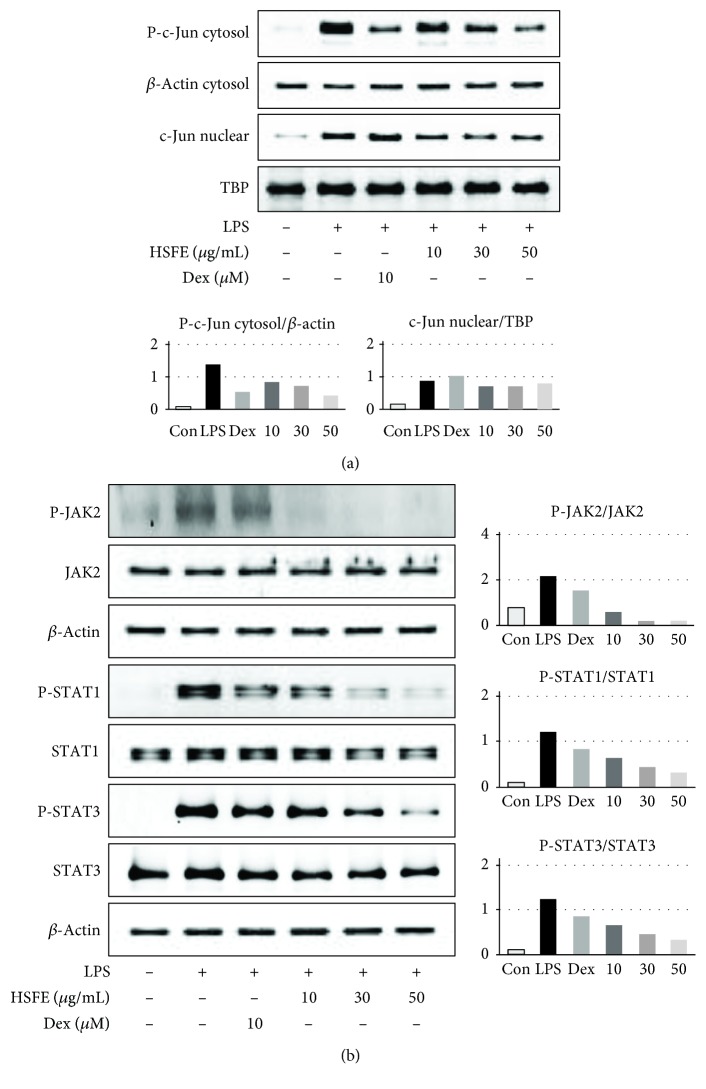
Effects of hoveniae semen seu fructus ethanol (HSFE) extract on (a) phosphorylation and nuclear translocation of c-Jun and (b) phosphorylation of JAK2/STAT1-3. Cells were seeded with 1.5 × 10^6^ cells/well on a 6-well culture plate and preincubated for 18 h. Then, cells were pretreated with HSFE for 1 h and stimulated with LPS for another (a) 30 min or (b) 4 h. At least three independent tests were repeated to ensure reproducibility. *β*-Actin and TATA box-binding protein (TBP) served as the controls for cytosolic and nuclear proteins, respectively. Data in the histograms show protein expression levels relative to *β*-actin or TBP. The results shown are representative of three separate experiments. Con: control.

AP-1 is another important transcription factor and is linked to the production of several proinflammatory mediators [9]. AP-1 migrates into the nucleus and regulates expression of certain inflammatory genes when macrophages become inflammatory in response to conditions caused by stimuli, such as LPS. We therefore measured the level of phosphorylation in the cytoplasm and the amount transferred into the nucleus of the AP-1 subunit c-Jun protein.  Figure 5(a) shows LPS stimulation-related phosphorylation and migration into the nucleus of the c-Jun protein, whereas HSFE pretreatment effectively and strongly inhibited nuclear transfer and phosphorylation of c-Jun.

### 3.6. Effect of HSFE on Activation of the JAK/STAT Signaling Pathway in LPS-Stimulated RAW 264.7 Cells

Several previous studies have reported that mitigation of the JAK/STAT signaling pathway inhibited LPS-induced NO and proinflammatory cytokine production [20]. Therefore, we measured the HSFE effect on phosphorylation of JAK2, STAT1, and STAT3 in RAW 264.7 cells stimulated with LPS by Western blot. As shown in Figure 5(b), HSFE treatment significantly blocked phosphorylation of JAK2, STAT1, and STAT3 at concentrations of 30–50 *μ*g/mL and did not affect the total protein level. These results indicate that HSFE treatment not only inhibited phosphorylation and nuclear transcription of the c-Jun protein but also inhibited JAK/STAT pathway activation.

### 3.7. Effect of HSFE on p65 Translocation in LPS-Stimulated RAW 264.7 Cells

**Figure 6:**
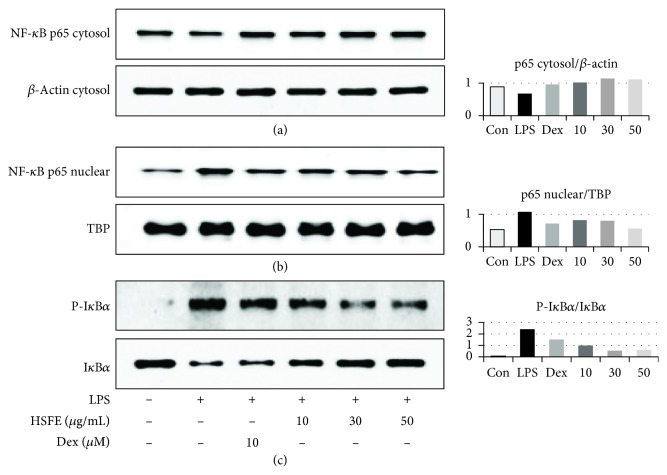
Effects of hoveniae semen seu fructus ethanol (HSFE) extract on (a, b) nuclear translocation of NF-*κ*B p65 and (c) degradation of I*κ*B*α*. Cells were seeded with 1.5 × 10^6^ cells/well on a 6-well culture plate and preincubated for 18 h. Then, cells were pretreated with HSFE for 1 h and stimulated with LPS for another (a, b) 1 h or (c) 30 min. At least three independent tests were repeated to ensure reproducibility. *β*-Actin and TBP served as the controls for cytosolic and nuclear proteins, respectively. Data in the histograms show protein expression levels relative to those of *β*-actin, TBP, or I*κ*B*α*. The results shown are representative of three separate experiments. Con: control.

The transcription factor NF-*κ*B is a pivotal regulator closely associated with inflammatory responses. Thus, we examined whether HSFE inhibits LPS-induced p65 translocation and inhibition of NF-*κ*B alpha (I*κ*B*α*) phosphorylation. Our data show that HSFE exclusively blocks p65 translocation in the nucleus in a concentration-dependent manner (Figures 6(a) and 6(b)). Additionally, as seen in the Western blot analysis, HSFE inhibits degradation and activation of I*κ*B*α* by LPS stimulation (Figure 6(c)) in a concentration-dependent manner. These results suggest that HSFE reduces the translocation of the NF-*κ*B subunit p65 by preventing the I*κ*B*α* degradation.

### 3.8. Effect of HSFE on LPS-Induced Cytokine Levels in Mouse Peritoneal Macrophages

**Figure 7:**
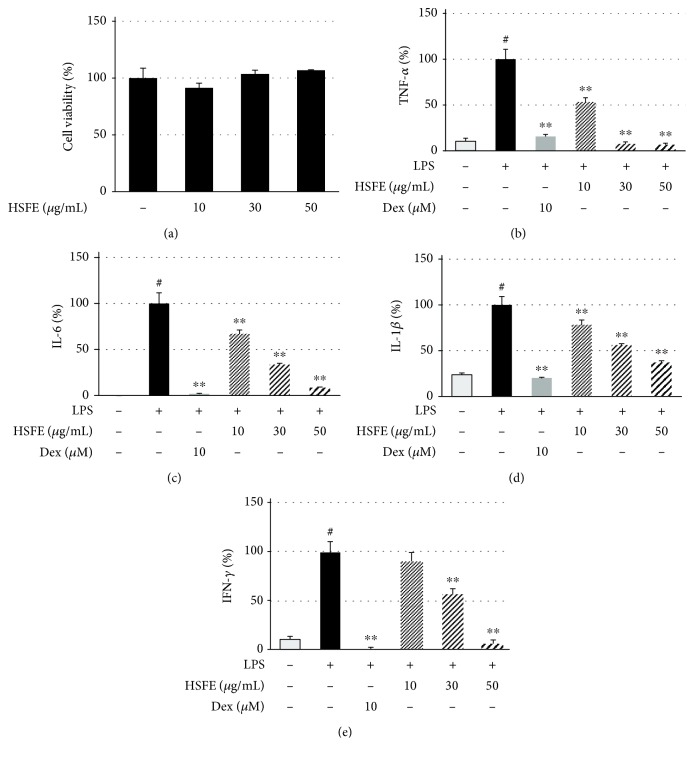
Effects of hoveniae semen seu fructus ethanol (HSFE) extract on (a) cell viability and production of the inflammatory cytokines (b) TNF-*α*, (c) IL-6, (d) IL-1*β*, and (e) IFN-*γ* in mouse peritoneal macrophages. The primary macrophages obtained from 6 BALB/c mice were seeded with (a) 5.0 × 10^4^ cells/well on a 96-well culture plate or (b–e) 2.5 × 10^5^ cells/well on a 24-well culture plate and preincubated for 18 h. Then, cells were pretreated with HSFE for 1 h and then stimulated with LPS for another 24 h. At least three independent tests were repeated to ensure reproducibility of the experimental results. As a control, cells were incubated with vehicle alone. Data represent the mean ± SEM of duplicate determinations from three independent experiments. ^#^*P* < 0.001 (vs. control) and ^∗∗^*P* < 0.001 (vs. LPS) values were considered statistically significant.

To confirm the anti-inflammatory efficacy of HSFE in mouse primary macrophages, we also measured inflammatory cytokine levels in LPS-stimulated mouse peritoneal macrophages via ELISA. HSFE pretreatment did not affect the cell viability of the primary macrophages (Figure 7(a)) and strongly inhibited TNF-*α*, IL-6, IL-1*β*, and interferon- (IFN-) *γ* cytokine secretion in a dose-dependent manner (Figures 7(b)–7(e)); additionally, each cytokine secretion was inhibited by 93%, 91%, 63%, and 95%, respectively, at the 50 *μ*g/mL concentration of HSFE treatment.

### 3.9. HPLC Analysis of HSFE

**Figure 8:**
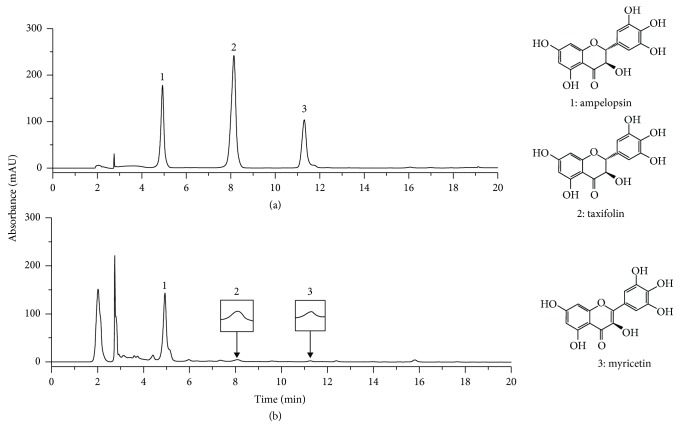
HPLC chromatograms of (a) three standard mixtures and (b) HSFE at 280 nm. 1: ampelopsin; 2: taxifolin; 3: myricetin. (a) Standard compound: 280 nm, (b) HSFE: 280 nm.

The constituents of HSFE were determined by HPLC analysis, and each peak of UV spectra was compared with that of representative standard compounds. As described in Figure 8(a), at the 280 nm UV detection wavelength, the retention times of ampelopsin, taxifolin, and myricetin in the standard mixture were 4.93, 8.14, and 11.30 min, respectively. Under the same conditions, the retention times of the observed components were 4.93, 8.07, and 11.26 min in HSFE, respectively (Figure 8(b)).

### 3.10. Verification of Anti-Inflammatory Efficacy of Ampelopsin, Taxifolin, and Myricetin in LPS-Stimulated RAW 264.7 Macrophages

**Figure 9:**
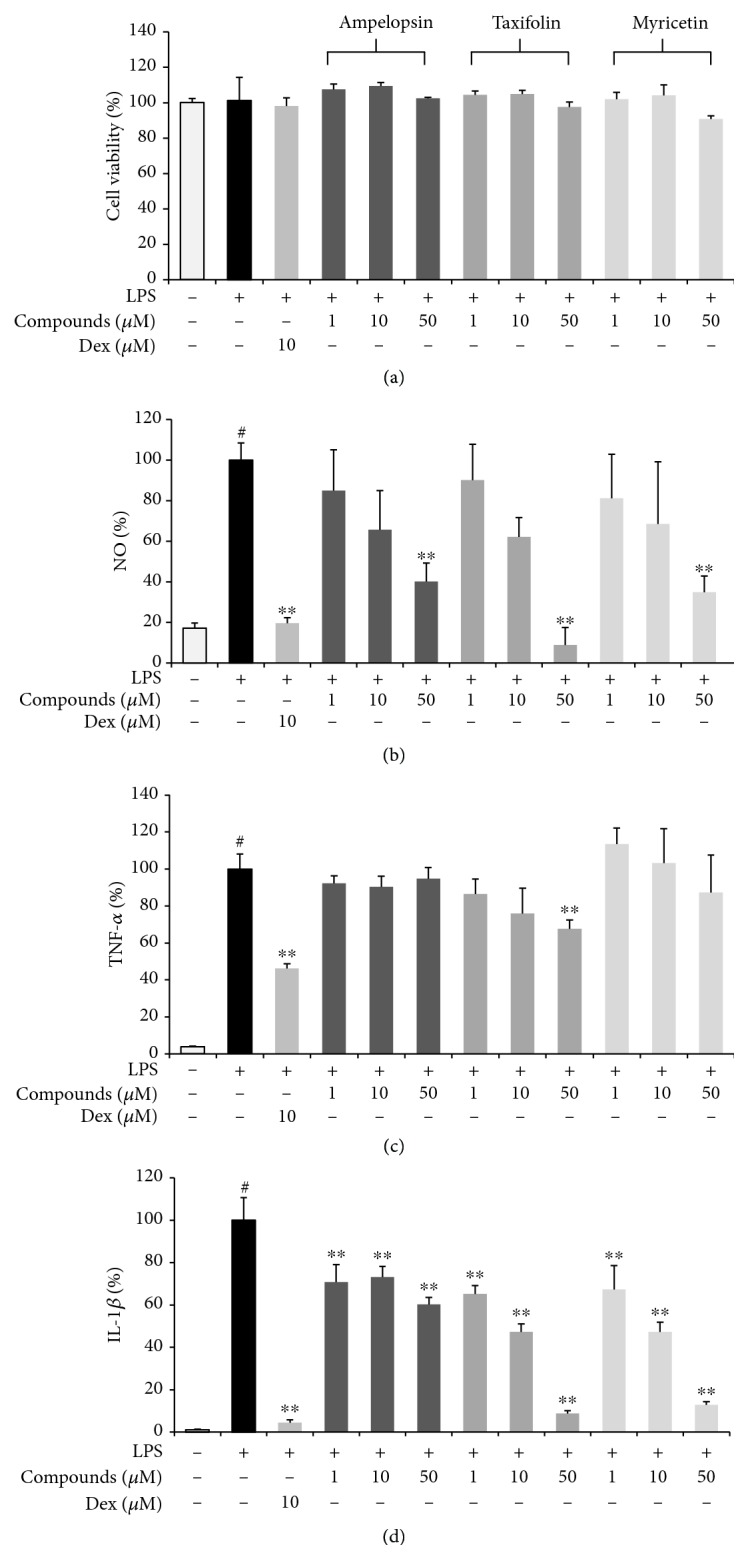
Effects of three compounds, ampelopsin, taxifolin, and myricetin, on (a) cell viability and the secretion of (b) NO and (c–e) inflammatory cytokines. Cells were seeded with (a, b) 5.0 × 10^4^ cells/well on a 96-well culture plate or (c–e) 2.5 × 10^5^ cells/well on a 24-well culture plate and preincubated for 18 h. Then, cells were pretreated with each compound for 1 h and stimulated with LPS for another 24 h. At least three independent tests were repeated to ensure reproducibility of the experimental results. (a) Cell viability was examined using a cell-counting kit. (b) NO secretion into the culture media was determined using the Griess assay. (c–e) Secretion of inflammatory cytokines was measured by ELISA. As a control, cells were incubated with vehicle alone. Data represent the mean ± SEM of duplicate determinations from three independent experiments. ^#^*P* < 0.001 (vs. control) and ^∗∗^*P* < 0.001 (vs. LPS) values were considered statistically significant.

The following experiments were carried out to confirm the inhibitory effects of the three major components of HSFE on macrophage inflammatory responses. First, the influence of the three compounds on the viability of macrophages was measured using a CCK. Ampelopsin and taxifolin showed no toxicity to macrophages at 1–50 *μ*M, whereas myricetin showed weak cytotoxicity at 50 *μ*M (survival of 90% or more) but did not affect the subsequent test (Figure 9(a)). Next, the effects of the three compounds on NO and inflammatory cytokine release in LPS-induced inflammatory responses were analyzed. Ampelopsin, taxifolin, and myricetin suppressed the secretion of NO in a concentration-dependent manner and showed a statistically significant inhibitory activity when each compound was pretreated at a high concentration (50 *μ*M) (Figure 9(b)). Only taxifolin showed a weak inhibitory activity against the inflammatory cytokine TNF-*α* at high concentration (50 *μ*M) (Figure 9(c)), and IL-6 secretion was relatively effectively repressed by all compounds in a concentration-dependent manner and was statistically significant (Figure 9(d)). IL-1*β* cytokine secretion was most effectively inhibited by pretreatment of the three compounds, with significant inhibition at all concentrations and increased efficacy in a concentration-dependent manner (Figure 9(e)).

## 4. Discussion

In previous studies, HSF has been reported to exhibit anti-inflammatory efficacy through blockade of the NF-*κ*B pathway in inflammatory macrophages. However, as potential therapeutic candidates, the effects of HSF on a variety of other signaling pathways involved in intracellular inflammatory responses and the scientific evidence remain unexplored. We therefore investigated the anti-inflammatory efficacy and other potential inhibitory mechanisms of HSFE in inflammatory-conditioned macrophages. In addition, the HSFE anti-inflammatory activity was confirmed in LPS-stimulated mouse primary macrophages. We first evaluated the effect of HSFE at ≤50 *μ*g/mL on the viability of macrophages and found that they did not show cytotoxicity, and subsequent experiments were performed at three concentrations. Next, the inhibitory activity of HSFE against NO production, the most basic parameter for evaluation of anti-inflammatory activity, was confirmed, and the effects on iNOS and COX-2 expressions were examined. iNOS and COX-2 are enzymes that synthesize NO and PGE_2_ from L-arginine and arachidonic acid, respectively, and are important targets in the study of anti-inflammatory agents [21, 22]. HSFE treatment had a superior inhibitory effect on iNOS expression, which demonstrated that the inhibitory effect on NO production was related to iNOS inhibition, but HSFE had no inhibitory effect on COX-2 expression. We also explored the efficacy of HSFE on secretion and mRNA gene expression of the proinflammatory cytokines, TNF-*α*, IL-6, and IL-1*β*, and proved that HSFE treatment exerted strong inhibitory activity against IL-6 and IL-1*β* production except against TNF-*α*.

To explore additional mechanisms of the HSFE anti-inflammatory activity, we investigated the effects of HSFE on activation of the MAPK, AP-1, and JAK/STAT signaling pathways by LPS stimulation. In macrophages, MAPK and AP-1 are important regulators of various genes of encoded inflammatory factors [23], MAPK has a vital role in signal transduction pathways in controlling immune responses and inflammatory factors [24], and MAPK phosphorylation after LPS stimulation is known to regulate AP-1 activation [7, 25]. AP-1 is an essential transcription factor consisting of the c-Jun/c-Fos heterodimer and is present in the cytoplasm as a quiescent form in unstimulated cells [26]. By inflammatory stimulation, AP-1 is activated and migrates into the nucleus, which promotes the production of proinflammatory mediators [27, 28]. Therefore, we first assessed the efficacy of HSFE on activation of the MAPK proteins ERK, p38, and JNK by LPS stimulation, which demonstrated that HSFE pretreatment effectively inhibited phosphorylation of each MAPK protein. In addition, we examined the effect of HSFE on intracellular phosphorylation levels and nuclear transfer of c-Jun protein by LPS treatment to measure the effect on AP-1 activation and demonstrated that HSFE treatment strongly inhibited both phosphorylation and nuclear translocation of AP-1. These results indicated that the anti-inflammatory activity of HSFE was due not only to blockade of the NF-*κ*B pathway known in previous studies but also to inhibition of activation of the MAPK and AP-1 pathways.

As an inflammatory pathway, the JAK/STAT pathway is known to elicit production of multiple inflammatory factors through phosphorylation and is recognized as having a central role in the immune and inflammatory responses [9, 29]. The JAK/STAT signaling pathway is activated by stimuli, such as LPS, to induce phosphorylation of downstream molecules, such as STAT1, which results in the translocation into the nucleus and binding to promoter regions of various proinflammatory mediators [30]. In addition, the activation of STAT3 has a direct effect on the IL-6 cytokine secretion [31]. Therefore, we measured whether the anti-inflammatory effect of HSFE in macrophages is associated with the inhibition of phosphorylation of the JAK2/STAT1-3 proteins, and as a result, HSFE treatment effectively inhibited phosphorylation of JAK2, STAT1, and STAT3. These results showed that HSFE treatment exerted anti-inflammatory activity by controlling the inflammatory response through inhibition of JAK/STAT activity in macrophages. In the case of natural products, the efficacy may vary depending on conditions, such as cultivated land and climate, so we have reexamined the inhibitory effect of HSFE on NF-*κ*B activation, which has been studied previously, and confirmed its robust activity.

Finally, we confirmed the anti-inflammatory activity of HSFE in LPS-stimulated mouse primary macrophages. As in the cell line, HSFE treatment did not show cytotoxicity at concentrations of <50 *μ*g/mL and effectively inhibited production of TNF-*α*, IL-6, IL-1*β*, and IFN-*γ* cytokines at all three concentrations. One peculiarity is that, unlike in RAW 264.7 cell lines, HSFE pretreatment in the LPS-stimulated mouse primary macrophages had a potent and dose-dependent inhibitory effect on secretion of TNF-*α*.

As shown in Figure 8, we identified three main components of HSFE (ampelopsin, taxifolin, and myricetin) consistent with previous studies [19]. Previous studies have shown that ampelopsin attenuates the inflammatory response by inhibiting NF-*κ*B and JAK/STAT signaling pathways in LPS-stimulated BV2 microglia [32] and suppresses inflammation by blocking the activation of PI3K/Akt/NF-*κ*B in RAW 264.7 macrophages [33]. In another study, taxifolin repressed NF-*κ*B *via* upregulation of the nuclear factor erythroid 2-related factor 2 (Nrf-2) pathway in experimental colon carcinogenesis [34], and myricetin inhibited the early inflammatory response by LPS [35]. In addition, recent studies have shown that myricetin inhibits Akt, mTOR, and NF-*κ*B in human keratinocytes, thereby reducing the expression of inflammatory factors [36], and exhibits anti-inflammatory activity through inhibition of NF-*κ*B, STAT1 activation, and HO-1 induction in RAW 264.7 macrophages [37]. These previous results indicate that the anti-inflammatory effect of HSFE is closely related to the activities of the three components (ampelopsin, taxifolin, and myricetin). In connection with the above, to confirm the anti-inflammatory activity of the main components of HSFE, we measured the inhibitory activities of ampelopsin, taxifolin, and myricetin on LPS-induced secretion of NO and inflammatory cytokines, including TNF-*α*, IL-6, and IL-1*β*, in RAW 264.7 macrophages. As a result, all three compounds showed effective anti-inflammatory activity in a concentration range that is not toxic to macrophages.

Considering the results of this study, HSFE has excellent control over inflammatory reactions at the *in vitro* level. HSFE has been used in the treatment of indirectly related diseases, although it is not a specialized herbal drug for inflammatory diseases in traditional medicine in East Asia, and the findings of this study suggest scientific evidence for this traditional theory. Based on the results, HSFE can be considered as a potential candidate for the prevention or treatment of inflammatory diseases in clinical applications after further *in vivo* study.

In summary, our study demonstrated that HSFE treatment suppressed the release of NO, iNOS, and inflammatory cytokines in RAW 264.7 macrophages stimulated by LPS and its efficacy was due to the inhibition of MAPK phosphorylation and blocking of AP-1, JAK2/STAT, and NF-*κ*B activation. In addition, our study showed that HSFE inhibited secretion of TNF-*α*, IL-6, IL-1*β*, and IFN-*γ* cytokines in mouse primary macrophages. Also, the anti-inflammatory efficacy of HSFE seems to be closely related to the presence of three main components (ampelopsin, taxifolin, and myricetin). These findings provide a novel insight into the anti-inflammatory activity of HSFE and its molecular mechanism of action.

## Figures and Tables

**Table 1:** Primary and secondary antibodies used for Western blot analysis.

Antibody	Corporation	Product no.	Dilution rate
iNOS	Cell Signaling Technology	#2977	1 : 1,000
COX-2	Cell Signaling Technology	#4842	1 : 5,000
*β*-Actin	Santa Cruz Biotechnology	#SC-47778	1 : 5,000
P-ERK	Cell Signaling Technology	#4377	1 : 1,000
ERK	Cell Signaling Technology	#9102	1 : 1,000
P-p38	Cell Signaling Technology	#9211	1 : 1,000
p38	Cell Signaling Technology	#9212	1 : 1,000
P-JNK	Cell Signaling Technology	#9251	1 : 1,000
JNK	Cell Signaling Technology	#9252	1 : 1,000
P-c-Jun	Cell Signaling Technology	#9164	1 : 1,000
c-Jun	Cell Signaling Technology	#9165	1 : 1,000
TBP	Cell Signaling Technology	#8515	1 : 1,000
P-JAK2	Santa Cruz Biotechnology	#SC-21870	1 : 500
JAK2	Santa Cruz Biotechnology	#SC-294	1 : 500
P-STAT1	Cell Signaling Technology	#7649	1 : 1,000
STAT1	Cell Signaling Technology	#9172	1 : 1,000
P-STAT3	Cell Signaling Technology	#9361	1 : 1,000
STAT3	Cell Signaling Technology	#9139	1 : 1,000
NF-*κ*B p65	Cell Signaling Technology	#3034	1 : 1,000
P-I*κ*B*α*	Cell Signaling Technology	#2859	1 : 1,000
I*κ*B*α*	Cell Signaling Technology	#4814	1 : 1,000
Secondary anti-mouse	Cell Signaling Technology	#7076	1 : 5,000
Secondary anti-rabbit	Cell Signaling Technology	#7074	1 : 5,000

**Table 2:** Primers used for real-time RT-PCR.

Target gene	Primer sequence
TNF-*α*	F: 5′-TTCTGTCTACTGAACTTCGGGGTGATCGGTCC-3′
	R: 5′-GTATGAGATAGCAAATCGGCTGACGGTGTGGG-3′

IL-6	F: 5′-TCCAGTTGCCTTCTTGGGAC-3′
	R: 5′-GTGTAATTAAGCCTCCGACTTG-3′

IL-1*β*	F: 5′-ATGGCAACTGTTCCTGAACTCAACT-3′
	R: 5′-CAGGACAGGTATAGATTCTTTCCTTT-3′

iNOS	F: 5′-GGCAGCCTGTGAGACCTTTG-3′
	R: 5′-GCATTGGAAGTGAAGCGTTTC-3′

COX-2	F: 5′-TGAGTACCGCAAACGCTTCTC-3′
	R: 5′-TGGACGAGGTTTTTCCACCAG-3′

HO-1	F: 5′-TGAAGGAGGCCACCAAGGAGG-3′
	R: 5′-AGAGGTCACCCAGGTAGCGGG-3′

*β*-Actin	F: 5′-AGAGGGAAATCGTGCGTGAC-3′
	R: 5′-CAATAGTGATGACCTGGCCGT-3′

F: forward; R: reverse.

## Data Availability

The data used to support the findings are available from the corresponding author upon request.
